# Spatial-temporal analysis of pulmonary tuberculosis in the northeast of the Yunnan province, People’s Republic of China

**DOI:** 10.1186/s40249-017-0268-4

**Published:** 2017-03-24

**Authors:** Li Huang, Xin-Xu Li, Eniola Michael Abe, Lin Xu, Yao Ruan, Chun-Li Cao, Shi-Zhu Li

**Affiliations:** 1 National Institute of Parasitic Diseases, Chinese Center for Disease Control and Prevention; Key Laboratory of Parasite and Vector Biology, Ministry of Health, WHO Collaborating Center for Tropical Diseases, Shanghai, 200025 People’s Republic of China; 2 Tuberculosis Program, Yunnan Center for Disease Control and Prevention, 158 Dongsi Road, Xishan District, Kunming, Yunnan 650022 People’s Republic of China; 3 National Center for Tuberculosis Control and Prevention, Chinese Center for Disease Control and Prevention, Beijing, 102206 People’s Republic of China

**Keywords:** Tuberculosis, Pulmonary Tuberculosis, Space-time clusters, Yunnan, China

## Abstract

**Background:**

The number of pulmonary tuberculosis (PTB) cases in China ranks third in the world. A continuous increase in cases has recently been recorded in Zhaotong prefecture-level city, which is located in the northeastern part of Yunnan province. This study explored the space-time dynamics of PTB cases in Zhaotong to provide useful information that will help guide policymakers to formulate effective regional prevention and control strategies.

**Methods:**

The data on PTB cases were extracted from the nationwide tuberculosis online registration system. Time series and spatial cluster analyses were applied to detect PTB temporal trends and spatial patterns at the town level between 2011 and 2015 in Zhaotong. Three indicators of PTB treatment registration history were used: initial treatment registration rate, re-treatment registration rate, and total PTB registration rate.

**Results:**

Seasonal trends were detected with an apparent symptom onset peak during the winter season and a registration peak during the spring season. A most likely cluster and six secondary clusters were identified for the total PTB registration rate, one most likely cluster and five secondary clusters for the initial treatment registration rate, and one most likely cluster for the re-treatment registration rate. The most likely cluster of the three indicators had a similar spatial distribution and size in Zhenxiong County, which is characterised by a poor socio-economic level and the largest population in Yunnan.

**Conclusion:**

This study identified temporal and spatial distribution of PTB in a high PTB burden area using existing health data. The results of the study provide useful information on the prevailing epidemiological situation of PTB in Zhaotong and could be used to develop strategies for more effective PTB control at the town level. The cluster that overlapped the three PTB indicators falls within the geographic areas where PTB control efforts should be prioritised.

## Background

Tuberculosis (TB) is an infectious disease caused by the bacteria *Mycobacterium tuberculosis*. Pulmonary tuberculosis (PTB) is a result of an attack on the lungs by *Mycobacterium tuberculosis*. Tuberculosis continues to be a major public health problem in China with an estimated one million incident cases reported which alone contributed 11% to the global TB incident in 2010 [1]. Therefore, the Chinese national authorities established the 12th five-year National TB Control Programme in 2011 to effectively control TB. The implementation of the integrated control strategies has helped control the burden of PTB in China during the last 5 years. However, a continuous increase of the PTB burden has recently been observed in Yunnan province, especially in Zhaotong prefecture-level city.

The number of active PTB cases in Zhaotong is ranked highest in Yunnan and contributes almost one-quarter of the provincial registered cases yearly [2]. The epidemic disparity of PTB between Zhaotong and other cities in Yunnan appears to follow an increasing trend yearly. The notification rate of PTB in Zhaotong is twice and four times higher than the rate reported for the province and for Yuxi city, which has the lowest notification rate in Yunnan, respectively [2].

The use of spatial-temporal cluster analysis has been applied for the detection of infectious disease hotspots in recent years [3–5]. It has also been successfully used for the identification of TB clusters, with meaningful results yielded [6–8]. Previous studies have shown that PTB is an airborne infectious disease with spatial and temporal heterogeneous distribution, and it is believed that a better understanding of the spatial epidemiology of PTB will help guide policymakers and different stakeholders to formulate effective regional prevention and control strategies [9–11].

Until now, few studies have been conducted to explore the spatial epidemiology of TB in Yunnan, China. Therefore, we conducted a spatial-temporal cluster analysis using town-level PTB registered data from 2011 to 2015 in Zhaotong to determine the clustering areas of the PTB epidemic and provide evidence to the local TB control programme for strategy development and intensified intervention.

## Methods

### Study setting

Yunnan Province is located in southwest China and has a relatively poor socio-economic status. In 2015, the average gross domestic product per capita was 29 015 Yuan, and it was only 13 060 Yuan in Zhaotong City. There are 47 million inhabitants in Yunnan [12]. The province covers an area of 394 100 km^2^, of which 94% is mountainous. There are 16 prefecture-level cities in Yunnan with a total of 129 counties. Zhaotong, located in the northeast of Yunnan, is strategically located in the province as it borders both the Guizhou and Sichuan provinces. There are 5.43 million inhabitants in Zhaotong, covering an area of 23 021 km^2^, containing 11 counties and 143 towns, with 23 ethnic minorities living in the mountainous and hilly areas.

### Data collection and management

The data on TB cases were obtained from the database of the Yunnan provincial TB programme at the Yunnan Provincial Center for Disease Control and Prevention (Yunnan CDC). All the cases were reported to the nationwide TB online registration system. The epidemiological data of each case were collected, including: age, gender, current address, date of TB symptom onset, date of diagnosis, result of smear microscopy, therapeutic category and result of treatment. Town-level PTB registration data in Zhaotong from 2011 to 2015 were extracted from the database system. Three indicators of PTB treatment registration history were used: initial treatment registration rate, re-treatment registration rate and total PTB registration rate. All the cases were geocoded and matched to the town-level polygon maps from the geographic information system (Geographic database from China CDC) at a 1:1 000 000 scale as the layer’s attribute table by the same identified number.

Furthermore, the town-level point layer containing latitudes and longitudes of central points for each town was created using the ArcGIS v10.2 software (ESRI, Redlands, CA, USA). The geocoding process was applied, as described in previous studies [13–15]. Demographic information and the administrative code of Zhaotong were obtained from the Annual Statistical Report and integrated into the study database.

### Statistics analysis

#### Time series analysis and descriptive analysis

**Fig. 1:**
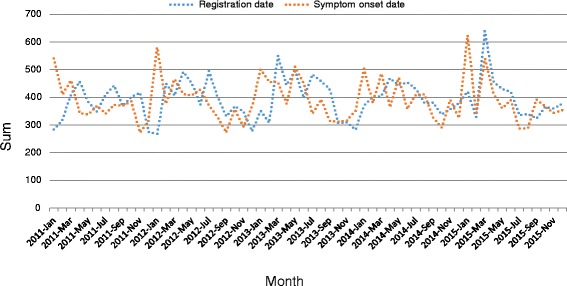
The monthly fluctuation of PTB cases in Zhaotong city, Yunnan province, China, from 2011 to 2015

All the PTB cases were aggregated by month in relation to their registration date and their symptom onset date, to identify the temporal patterns of the disease. The time series included 60 months in total from January 2011 to December 2015, and was examined using the EXCELL 2007 (see Fig. 1). The gender, age and the year of infection of the PTB cases were also aggregated to identify the demographic characteristics of the disease yearly. Time series and descriptive analyses were conducted at the town level as all the PTB cases registered in Zhaotong during the period between 2011 and 2015 were included in our analyses.

#### Spatial and space-time scan statistic

The spatial and space-time scan statistic was performed using SaTScan^TM^ software v9.4.2 [16]. The purely spatial scan statistic was applied to identify geographic areas with high PTB registration rates that statistically significantly exceeded nearby areas. The standard purely spatial scan statistic imposes a circular window on the map. The space-time scan statistic is defined by a cylindrical window with a circular geographic base and with height corresponding to time. The base is defined as the purely spatial scan statistic, while the height reflects the time period of potential clusters. The cylindrical window is then moved in space and time, so that for each possible geographical location and size, it also visits each possible time period. For this analysis, a Poisson based model was used, where the number of events in an area is Poisson distributed according to a known underlying population at risk. PTB cases registered in each town were used and recorded with the population in the same town which was assumed as the population in the Poisson probability model. Under the null hypothesis, and when there are no covariates, the expected number of cases in each area is proportional to its population size, or to the person-years in that area. For this analysis, the geographic size of the window was limited to half the expected number of cases and the time period was also limited to half the total time period. The test of significance of the identified clusters was based on comparing the likelihood ratio test statistics against a null distribution obtained from a Monte Carlo simulation. The number of permutation was set to 999 and the significance level was set as 0.05. The space-time scan window with the maximum likelihood value was the most likely cluster and other significant windows were called secondary clusters [17].

## Results

### Descriptive analysis of PTB cases

A total of 23 605 confirmed active PTB cases were included in this study, of which 21 lived in other provinces. Table 1 shows that the number of male cases was twice that of female cases in any given year. In addition, significant shares of PTB infections were found in the age groups of 15–30 years (over 30%) and 30–45 years (around 25%). Among the registered cases, the percentage of sputum smear-positive (SS+) PTB cases declined yearly (see Table 1).

**Table 1:** The demographic characteristics of PTB cases in Zhaotong, China from 2011 to 2015

	2011	2012	2013	2014	2015	Total
Gender						
Male	3 113(68.93)	3 179(68.17)	3 245(67.38)	3 273(68.03)	3 199(66.66)	16 009(67.82)
Female	1 403(31.07)	1 484(31.83)	1 571(32.62)	1 538(31.97)	1 600(33.34)	7 596(32.18)
Age						
0–15 year	105(2.33)	120(2.57)	108(2.24)	118(2.45)	210(4.38)	661(2.80)
15–30 year	1 570(34.77)	1 581(33.91)	1 732(35.96)	1 771(36.81)	1 723(35.90)	8 377(35.49)
30–45 year	1 184(26.22)	1 231(26.40)	1 272(26.41)	1 198(24.90)	1 145(23.86)	6 030(25.55)
45–60 year	984(21.79)	1 036(22.22)	1 004(20.85)	1 012(21.04)	991(20.65)	5 027(21.30)
> 60 year	673(14.90)	695(14.90)	700(14.53)	712(14.80)	730(15.21)	3 510(14.87)
Diagnosis						
Sputum smear-positive (SS+) TB	2 177(48.21)	1 439(30.86)	1 177(24.44)	864(17.96)	761(15.86)	6 418(27.19)
Sputum smear-negative(SS-)TB	2 224(49.25)	3 094(66.35)	3 470(72.05)	3 800(78.99)	3 804(79.27)	16 392(69.44)
Sputum smear not done	1(0.02)	0(0.00)	0(0.00)	6(0.12)	12(0.25)	19(0.08)
Tuberculosis pleurisy cases	114(2.52)	130(2.79)	169(3.51)	141(2.93)	222(4.63)	776(3.29)
Therapeutic Category						
Initial treatment	4 076(90.26)	4 409(94.55)	4 549(94.46)	4 638(96.40)	4 692(97.77)	22 364(94.74)
Retreatment	440(9.74)	254(5.45)	267(5.54)	173(3.60)	107(2.23)	1 241(5.26)

### Temporal patterns of PTB cases

Figure 1 shows the monthly time series of the numbers of PTB cases registered from 2011 to 2015. There was an evident trend variation; with the maximum number of PTB cases registered in the spring each year and a volatile declining trend the following summer and autumn, and then a minimum number of cases were recorded in winter. Peaks of the PTB symptom onset date were observed every January from 2011 to 2015, and then declined gradually after March. A non-linear correlation was observed both in the registration date (*χ* ^2^ = 336.831, *P* < 0.001) and symptom onset date (*χ* ^2^ = 6.703, *P* = 0.010), when comparing the different seasons amalgamated across all years (see Fig. 1).

### Spatial patterns of PTB cases

**Fig. 2:**
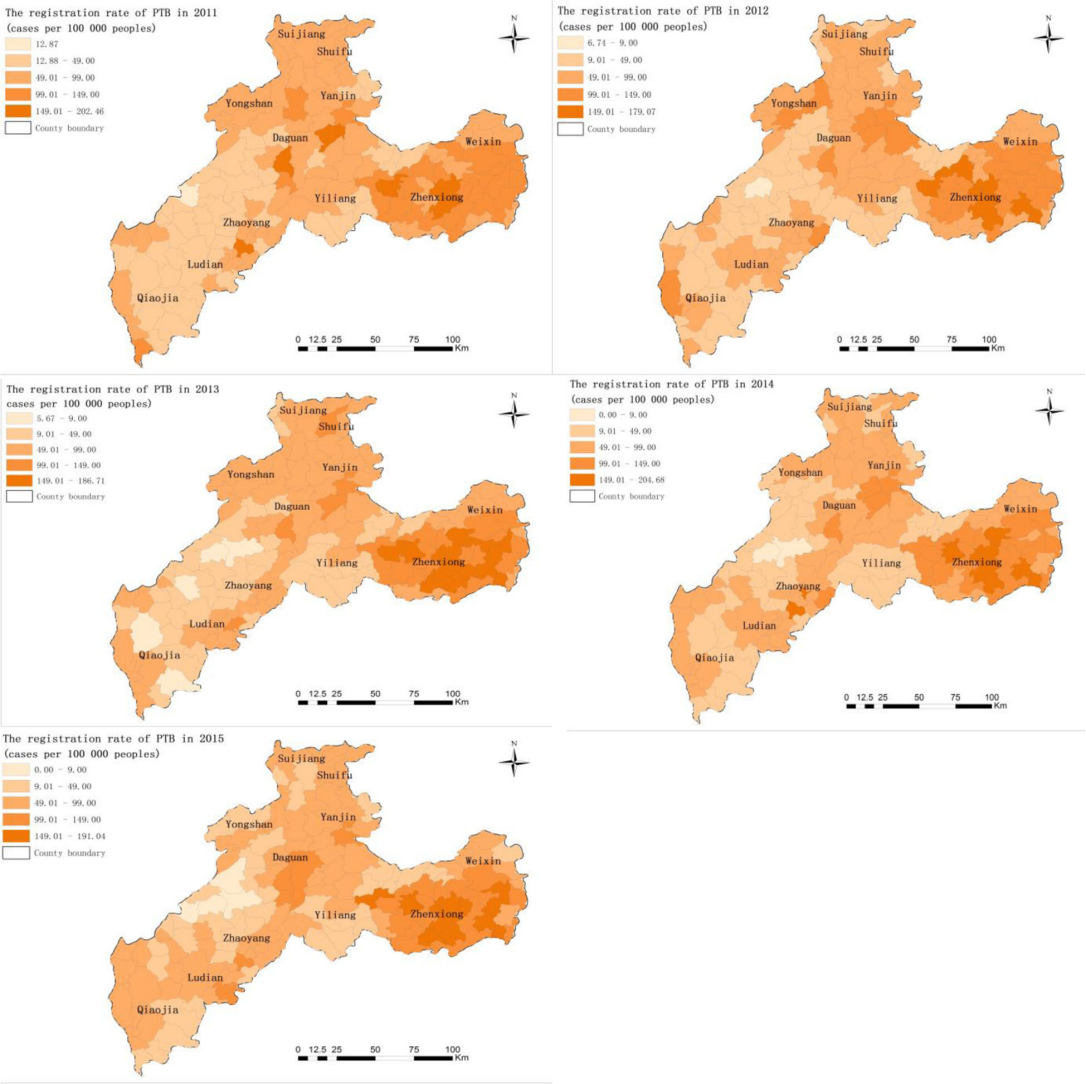
The registration rate of the total PTB cases (the number of PTB cases per 100 000 population) at the town level in Zhaotong, 2011–2015. Wuzhai town and Makou town did not record any PTB cases, respectively, in 2014 and 2015

The spatial variations of total PTB registration rates between 2011 and 2015 in Zhaotong shows that the annualised average rate at the town level ranged from 8.09 to 175.50 per 100 000 people. The highest registration rates were found in the towns of Zhenxiong County and border towns in Weixin County, such as Shuitian, Linfeng and Shuanghe (Fig. 2). In addition, Yongfeng and Xiaolongdong towns in Zhaoyang district; Yanjing, Dousha and Shizi towns in Yanjin County; and Cuihua town in Daguan County had the highest registration rates. The rates were relatively low in Yongshan County, Qiaojia County and Yiliang County. The cut-off values were the same, which is a visual display to show the registration rate of each town changes over time. The registration rates of PTB cases were significantly different during the years under study, and the registration rate in Wufeng town in Zhenxiong County appeared to increase from 148.94 in 2011 to 191.03 per 100 000 people in 2015. The disease demonstrated high heterogeneity in space and time.

**Fig. 3:**
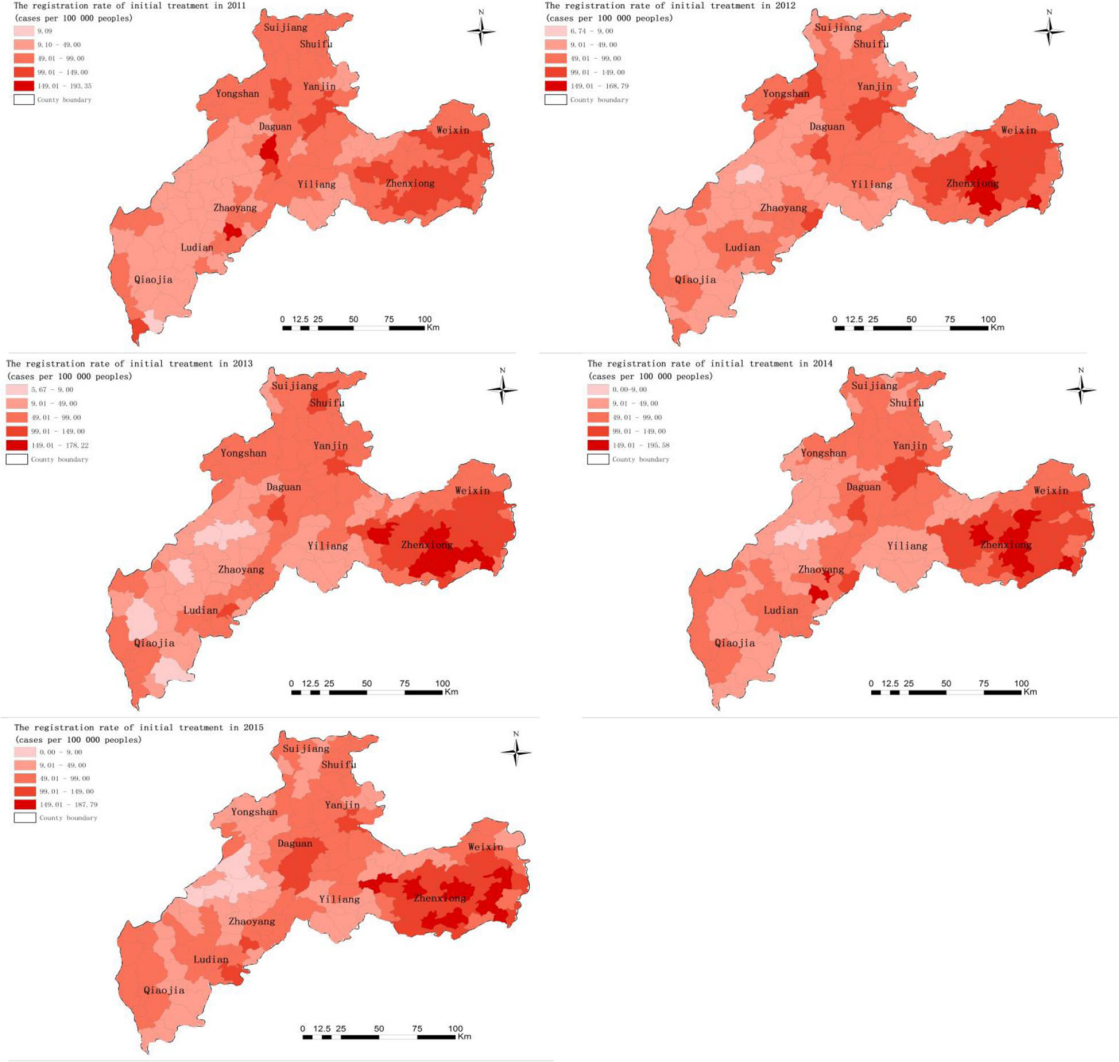
The registration rate of initial PTB treatment cases (the number of initial treatment cases per 100 000 population) at the town level in Zhaotong, 2011–2015

The spatial variations of initial treatment registration rates between 2011 and 2015 in Zhaotong shows that the annual cases per 100 000 people were 76.8. The highest registration rates were found in Wufeng, Yudong, Wanchang and Chishuiyuan towns in Zhenxiong County and Yongfeng town in Zhaoyang County (Fig. 3). The registration rates of initial treatment cases were significantly different between 2011 and 2015, while the rates in Wufeng town and Yudong town in Zhenxiong County increased continuously between 2011 and 2015.

**Fig. 4:**
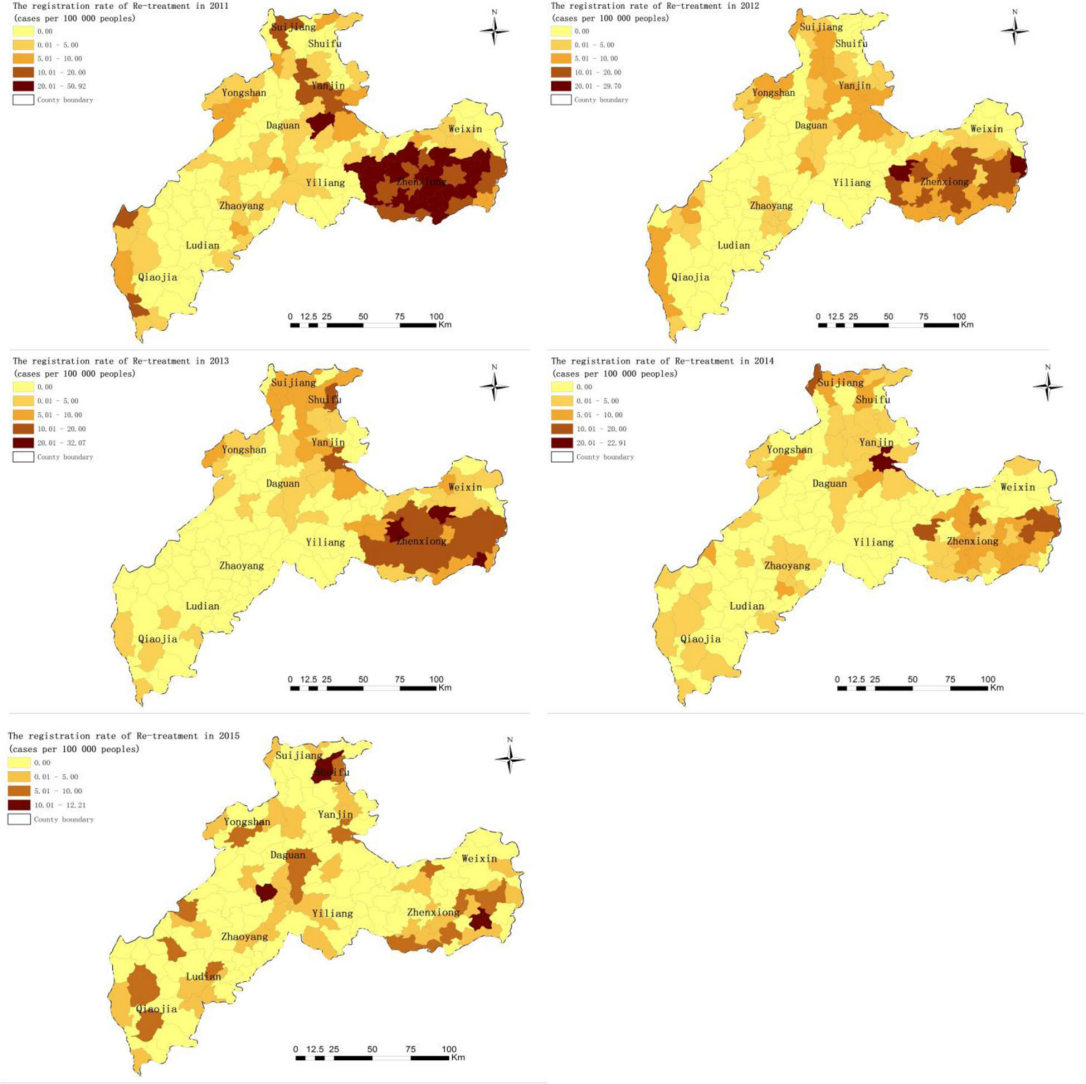
The registration rate of PTB re-treatment cases (the number of re-treatment cases per 100 000 population) at the town level in Zhaotong, 2011–2015

Figure 4 shows the spatial variations of PTB re-treatment registration rates between 2011 and 2015 in Zhaotong. Annual cases per 100 000 people were 4.3. The highest registration rates were found in Wanchang and Yanyuan towns of Zhenxiong County, Yanjing town in Yanjin County and Shanggaoqiao town in Daguan County. The registration rates of re-treatment cases were significantly different in the studied years. There was an obvious decrease in the registration of re-treatment rates in Wanchang and Yanyuan. However, the rate in Shanggaoqiao continuously increased from 2011 to 2015.

**Fig. 5:**
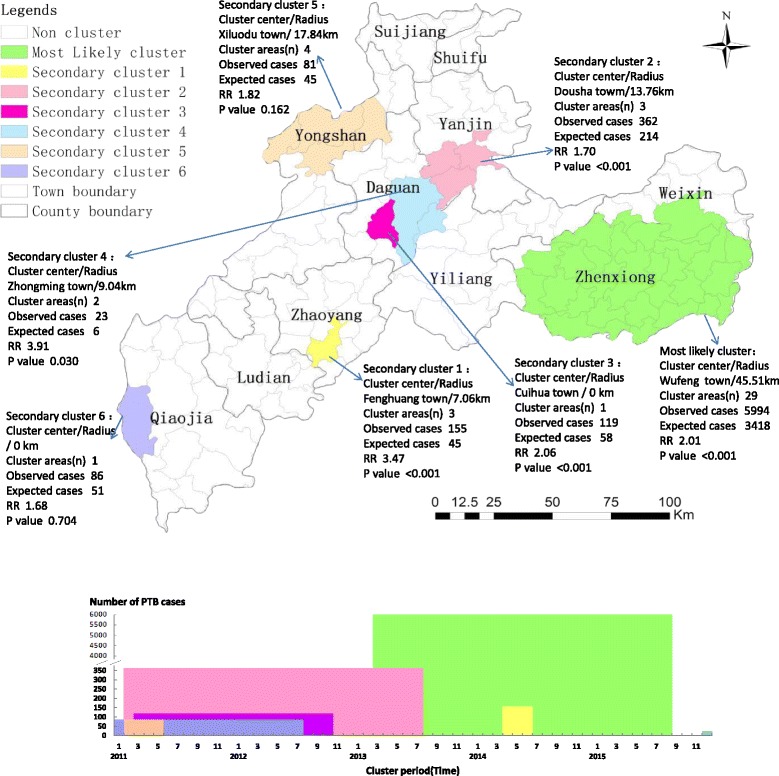
The space-time clusters of the total PTB cases at the town level in Zhaotong, 2011–2015. These space-time clusters are distinguished in different colours. The clusters that are statistically significant (*P* < 0.05) are labelled with their cluster centre and other details in the top panel. As show in the bottom panel, the height of the columns represents the total number of cases in the cluster across all the months in which cases in that cluster were reported

And the Fig. 5 shows the space-time scan analysis results regarding the total PTB cases using the Poisson model, including one most likely cluster and six secondary clusters. Two secondary clusters were statistically insignificant with *P* > 0.05. The clusters are distinguished in different colours on Fig. 5. The main cluster occupies large areas of the Zhenxiong County, covering 29 towns and 5 994 PTB cases. The cluster is significant, with long-term persistence observed from early spring 2013 to the end of summer 2015. The important finding is that people within this cluster were facing more than twice risk of acquiring PTB infection, compared to those outside the hotspot (relative risk, RR = 2.01). In addition, about three-quarters of the secondary clusters occurred in the spring and persisted for about 6 months. The other quarter of secondary clusters included Zhongming and LongAn towns in Yiliang County (secondary cluster 4) located in the south of Zhaotong, which emerged in December 2015 but lasted for a short period of only 1 month. The relative RR of these secondary clusters varied from 3.91 to 1.70.

**Fig. 6:**
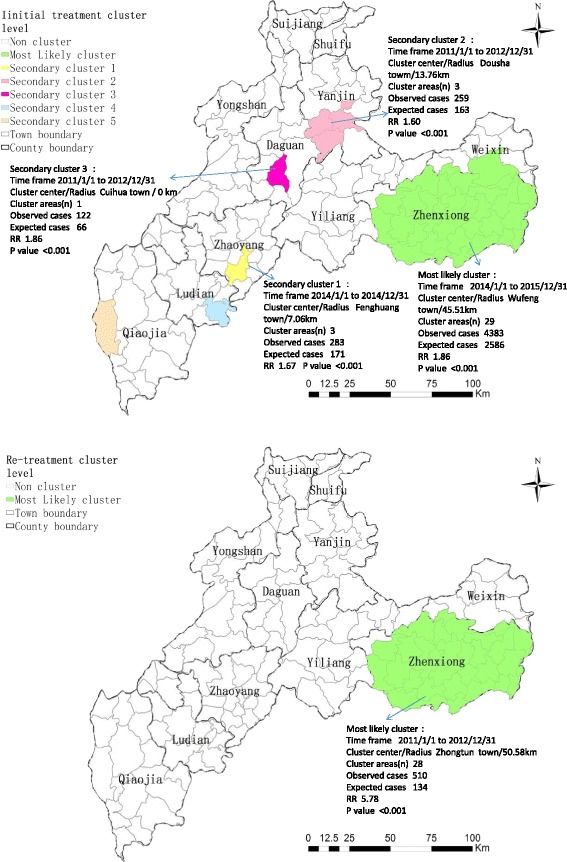
The space-time clusters of initial PTB treatment cases and re-treatment cases at the town level in Zhaotong, 2011–2015. The initial treatment clusters are shown in the top panel and the re-treatment cluster in the bottom panel

The space-time cluster for initial treatment and re-treatment cases was detected using a Poisson-based model. One most likely cluster and five secondary clusters for a high registration rate of initial treatment, and one most likely cluster for a high registration rate of re-treatment were detected, with the results depicted in Fig. 6. The most likely cluster of the PTB initial treatment registration rate had the same clustering centre and size as the total PTB registration rate in the Wufeng-central regions of Zhenxiong County (radius 45.51 km, covering 27 towns in Zhenxiong and two towns in Weixin County), between 2014 and 2015 (RR = 1.86, *P* < 0.001), with 4 383 observed cases and 2 586 expected cases. In addition, the significant secondary clusters of the initial treatment registration rate had the same clustering centres as the total PTB registration rate in Fenghuang, Dousha and Cuihua towns. The remaining two secondary clusters were statistically insignificant with *P* > 0.05. The most likely cluster for re-treatment cases was found to exist at Zhongtun town in Zhenxiong County (radius 50.58 km, covering 27 towns in Zhenxiong and Miaogou town in Weixin), between 2011 and 2012 (RR = 5.78, *P* < 0.001), with 134 observed cases and four expected cases.

## Discussion

This study analysed the spatial-temporal status of PTB in Zhaotong city, northeast Yunnan, using the smallest administrative unit in the city-wide scale. Hence, it is the first study to analyse PTB risk at the town level in a highly epidemic area of Yunnan. The identification of TB risk areas using surveillance data based on the geographic unit is advantageous for policymakers [18]. Systemic utilisation of cluster detection techniques for regular surveillance of TB is useful to effectively carry out TB control programmes [19]. Therefore, it is important to equip policymakers with information on the spatial-temporal status of PTB to help guide the provision, planning and optimisation of PTB control strategies in Zhaotong.

In this study, a total of 23 605 PTB cases were registered between 2011 and 2015 in Zhaotong. Findings show that there may be seasonal trends, with an apparent symptom onset peak in the winter and a registration peak during the spring of the following year. This reflects the delay between the time that PTB symptoms become evident in patients and the period that patients seek medical attention. It is also important to stress that most TB patients experience delay in referral due to their lack of awareness that there are designated PTB clinics and seek treatment in general hospitals. This might also lead to late PTB registration in our TB online registration system [20]. The possible reason of the symptom onset peak during the winter is a result of the reduction of exposure to ultraviolet rays from the sun and more indoor activities, which increases the possible susceptibility of people to infections with TB bacteria in the winter [21]. Similar trends in seasonal patterns had been reported elsewhere [22]. Resources should be distributed and planned for rationally in order to cope with the arrival of the incidence peak period and increase awareness-raising and running of focus groups during this period. It is of great significance to control the PTB epidemic situation and protect the elderly, patients with diabetes and chronic diseases and other susceptible groups.

The results obtained in this study show that the trend of the smear positive PTB cases percentage is very dramatic. The total number of smear positive cases decreased during the years under study, but there was a substantial increase in the number of smear-negative cases. The decrease in the number of smear-positive patients was due to the efforts for early case finding during the 12th five-year national TB integrated control plan that was initiated in 2011. Increased case finding efforts can result in a lower proportion of cases with bacteriological confirmation (that is smear positive), as some cases are found before the bacterial load increases to a detectable level [23]. Estimates from the World Health Organization Western Pacific Region lead to a similar conclusion: The proportion of cases bacteriologically confirmed among all TB cases decreased from 52% in 2005 to 37% in 2013 in this region [24].

This study identified total PTB space-time clusters, which differ in their socio-economic status, demography structures and natural environment. The most likely cluster included 27 towns in Zhenxiong County and two neighbouring towns in Weixin County, which have the highest risk of PTB infection (RR = 2.01). This region in Zhaotong is vast and densely populated, in which mostly poor people with very low incomes or without serious means of livelihood live. Therefore, a high prevalence of PTB infections in these areas is associated with a low socio-economic status, as poverty is reported to be one of the factors responsible for a high PTB prevalence. The prevalence of PTB infection in Zhaoyang district, which is ranked second to Zhenxiong County in terms of PTB burden in Zhaotong, as shown in the cluster, might be associated with the migration of people into the district. Zhaoyang is the capital of Zhaotong and also serves as the commercial centre of the region. The movement of PTB patients from the different counties could facilitate PTB transmission in the district.

The PTB spatial-temporal characteristics were identified using different indicators. As an indicator of PTB burden, the total PTB registration rate lumps together the initial treatment registration rate and the re-treatment registration rate. The initial treatment registration rate is one indicator of severe epidemic situation and the retreatment registration rate is one indicator of poor supervision and follow- up. The statistically significant clusters of initial treatment cases were in line with the total PTB cases. It is obvious that a high TB epidemic situation might trigger the development of initial treatment cases That is, people within the situation are more likely infected with TB bacteria than those of outside the situation. Thus, to achieve the target of reducing PTB cases, adequate attention should be given to the infected population in these clusters, and it is also important to optimise the use of available resources for effective PTB control. It is advised that the control of PTB should be incorporated into the public health services with standardised service contents and methods. These include: encouraging the role of health workers to disseminate information to patients in order to shorten the time interval between incidence and registration; and carrying out patients’ active screening in this cluster for early detection (case finding) – this should be incorporated into the control strategy plan for the next five years. In addition, more detailed survey-based investigations are needed within and outside the identified clusters to evaluate the factors influencing PTB distribution and purposeful intervention measures to control PTB prevalence in this region should be put forward.

The most likely cluster of the three indicators chosen for this study has similar spatial distribution and size in Zhenxiong County. This shows that the high registration rate of total PTB cases is not only limited to the high registration rate of initial treatment, but also the high registration rate of re-treatment. This area carries a disproportional burden of PTB cases. The overlap of clusters of the three indicators shows that there is a high active PTB incidence in this region, but patients do not receive adequate attention, desired care or satisfactory management. Therefore, there is a large number of re-treatment cases. One of the major challenges that might arise in this area is that if the management of patients cannot keep up with the numbers of PTB patients found, this might strongly influence the clustering of PTB re-treatment cases within a few years. Therefore, it is important to enhance treatment and management quality of active PTB cases under effective control strategy programmatic conditions in Zhenxiong County.

Though our study demonstrated the usefulness of spatial and temporal clustering analysis, it also had a few limitations. First, the estimated risk of PTB infection might be underestimated in some areas because cases may not be recorded in the official registration system. Second, the power of cylindrical scan statistics could be limited in some irregular geography. Third, we cannot judge the recent transmission or reactivation of PTB due to a lack of laboratory evidence, but we hope to improve this in future studies by accessing more funding to carry out molecular-based studies on TB transmission. Fourth, the potential risk factors that could be associated with clustering were not assessed in this study. The high PTB prevalence is frequently associated with individual and socio-economic factors [25–29]. Fifth, we did not get each patient’s geographic coordinates in this study. However, we can consider using individual coordinates in future studies when we analyse on village scale.

## Conclusion

Tuberculosis remains a public health burden in Zhaotong and our findings show that there are significant spatial-temporal characteristics of PTB at town scale in the region. Therefore, the findings of this study have provided useful information on the prevailing epidemiological situation of PTB in Zhaotong using existing health data and could be used to develop strategies for more effective PTB control at the town level.

## Abbreviations

CDC: Center of disease control and prevention
PTB: Pulmonary tuberculosis
RR: Relative risk
SS-: Sputum smear-negative
SS+: Sputum smear-positive
TB: Tuberculosis
